# Diet and supplement assessment in a Brazilian urban population

**DOI:** 10.11606/s1518-8787.2021055002356

**Published:** 2021-05-11

**Authors:** Alessandra Gaspar Sousa, Teresa Helena Macedo da Costa

**Affiliations:** I Universidade de Brasília Faculdade de Ciências da Saúde Programa de Pós-Graduação em Nutrição Humana BrasíliaDistrito Federal (DF) Brasil Universidade de Brasília. Faculdade de Ciências da Saúde. Programa de Pós-Graduação em Nutrição Humana. Brasília, Distrito Federal (DF), Brasil; II Centro Universitário Unieuro Departamento de Nutrição BrasíliaDistrito Federal (DF) Brasil Centro Universitário Unieuro. Departamento de Nutrição. Brasília, Distrito Federal (DF), Brasil; III Universidade de Brasília Faculdade de Ciências da Saúde Departamento de Nutrição BrasíliaDistrito Federal (DF) Brasil Universidade de Brasília. Faculdade de Ciências da Saúde. Departamento de Nutrição. Brasília, Distrito Federal (DF), Brasil

**Keywords:** Adult, Dietary Supplements, Nutritional Status, Body Mass Index, Exercise

## Abstract

**OBJECTIVE:**

To assess total usual nutrient intakes from foods and dietary supplements by age, sex, physical activity, and nutritional status, and to compare usual nutrient intakes to the Dietary Reference Intakes among non-users and users of dietary supplements in an urban population.

**METHODS:**

Cross-sectional population-based survey with 506 adults conducted in the city of Brasília, Brazil, using 24h food recalls. The 24-HR was collected on two nonconsecutive days, for which individuals reported all food, supplements, and beverages consumed in the previous 24 hours. The estimates of mean and the distribution percentiles were adjusted to reflect usual nutrient intake using the Iowa State University method. The prevalence of inadequate micronutrient intake was estimated according to sex using the Estimated Average Requirement (EAR), and values above the Tolerable Upper Intake Level (UL) were also considered. Also, a comparison was made of the total mean usual intake between supplement users and non-users according to BMI and physical activity.

**RESULTS:**

The total mean usual dietary intake was significantly higher among users than non-users of dietary supplements (p ≤ 0.02). Dietary supplement use increased intakes of nutrients and decreased prevalence of inadequacy according to sex, with only small (typically < 13%) increases in the population exceeding the Tolerable Upper Intake Level. There was a significant interaction between physical activity and BMI categories with supplement use.

**CONCLUSIONS:**

The population that consumes food supplements comprises individuals with more advanced age, female, normal BMI, and physically active. Our findings show that the use of supplements appears beneficial to attain nutrient adequacy. Careful monitoring of intake from food and supplements is recommended, and the statistical methods must be powerful enough to achieve relevant information.

## INTRODUCTION

Dietary supplements are commercially available products consumed in addition to the usual diet and include vitamins, minerals, herbs (botanicals), amino acids, and a variety of other products^1^. Dietary supplements intake has been shown to increase overall nutrient intake and decrease the prevalence of nutrient inadequacy^2^. Taking supplements seems to be a healthy and lifestyle choice, and the key motivators for consumers appear to be maintenance or improvement in overall health and specific health benefits rather than filling nutritional needs^2^^,^^3^. Nonetheless, the risks associated with dietary supplementation are well documented and include contamination of ingredients, inadvertent results, and undesirable side effects^2^^,^^4^.

The use of supplements in disease prevention and health promotion in epidemiological research is often difficult because supplement intake cannot be easily isolated from other healthy behaviors^2^. However, given the widespread use of dietary supplements, increased clinical research efforts are needed to address their safety and whether they are effective in compensating for dietary deficits^2^. Besides, many nutrients, notably vitamins and minerals, are derived from supplements as well as from foods and beverages. Thus, estimates of the total consumption of any nutrient must include supplement intake^5^.

In this sense, obtaining population data and correctly applying consumption methodologies become necessary to know the real magnitude of nutritional risk in our country; however, data on dietary supplement intake are insufficient. For instance, in the United States, the use of dietary supplements among adults has increased over the past 30 years. About 50% of adults currently take dietary supplements, and more than two-thirds of them use vitamin/mineral supplements^6^. In Brazil, a study carried out in the city of São Paulo showed that the prevalence of use of supplements was 6.35% and that higher consumption occurs among women^7^. However, no other study on the dietary intake of supplements at the population level in the Brazilian population has been identified.

Developed by the Food and Nutrition Board of the Institute of Medicine of the National Academy of Sciences, the Dietary Reference Intakes is the general term for a set of reference values (i.e., RDA, EAR, AI, UL etc.) used to plan and assess nutrient intakes. The Estimated Average Requirements (EAR) make it possible to estimate the prevalence of nutritional inadequacy in population groups. Additionally, the Tolerable Upper Intake Levels (UL) provide cutoff points for estimating the percentage of the population of interest at potential risk of adverse effects due to overconsumption of a nutrient. These two components of the DRIs provide opportunities for assessing nutrient intake and evaluating the influence of supplement use on dietary status^8^.

Although estimating usual nutrient intake distributions using food sources of nutrients alone is mostly solved, the same cannot be said for nutrient intake from supplement sources^9^. The challenge inherent in such a strategy lies in combining daily intake food/beverages with usual intake from supplements^5^. Thus, the collection of population data and the correct application of methodologies are necessary to understand the real magnitude of the nutritional risk. However, there is scarce data on dietary intake of supplements, especially in Brazil.

This study aims to assess the total usual nutrient intakes from foods and dietary supplements by age, sex, physical activity, and nutritional status and to compare usual nutrient intakes to the Dietary Reference Intakes among non-users and users of dietary supplements in an urban population.

## METHODS

### Study Design and Population

The study is a cross-sectional population-based survey conducted from February 2016 to July 2017. Sample determination was performed using a stratified sampling by clusters, considering as the primary unit of sampling the households proportionally distributed among the four sanitary regions of the central area of the city of Brasília, located in the region of the Federal District. The sample calculation was based on households registered with the Electric Power Company of Brasilia. The sample size was calculated using an alpha error of 5% and an 80% prevalence of physical activity below 150 minutes per week, which resulted in 250 households and at least 500 individuals, considering two adults per household^10^.

Out of 506 individuals, 363 (72%) were from the replacement sample, as detailed elsewhere^11^. In brief, sample replacement was done by recruiting the household following the sequence of the random household list, which contained 20% more addresses, or by invitation to residents of the same region, by telephone and social networks. The total number of households in our study was 429, keeping the proportion in each of the four Brasília areas.

The following exclusions were applied: visitors (characterized as individuals not actually living in the house but who were present at the moment of the interview) at the interviewed household, pregnant women, nursing mothers, people with disabilities, or intellectually disabled who were unable to describe their diet or diets that could not be measured using a scale and anthropometer. We interviewed residents aged ≥ 20 years who accepted our invitation.

This research protocol was approved by the Research Ethics Committee of the *Faculdade de Ciências da Saúde* of the *Universidade de Brasília* (Protocol n.1.350.858 CAAE 48418315.4.0000.0030).

### Data Entry and Databases

At the first visit to the household, a structured questionnaire was used to collect information about demographics (age, sex, socioeconomic classification, years of schooling, and physical activity). The practice of physical activity was also recorded and certified in a form encompassing the description of the physical activities of the last 24 hours. A 24-h dietary recall (24-HR) was applied, and anthropometric measurements were assessed according to the protocol of the WHO (1995)^12^. Weight and height were obtained to calculate the body mass index (BMI), and the WHO cutoffs were used to classify the respondents^12^.

The 24-HR was collected on two nonconsecutive days, for which individuals reported all food, supplements, and beverages consumed in the previous 24 hours. To collect information on the dietary supplement intake, the individuals were asked about the brand and the dosage of the supplements consumed in the previous 24 hours. The United States Department of Agriculture's (USDA) five-step multiple-pass method was followed^13^. The first 24-HR was conducted in person, and the second was conducted by telephone, during the week of the first interview or within a fortnight of the in-person interview.

The 24-HR food and portions data were translated to nutrients using the Nutrition Data System for Research software version 2016 (NDSR, Nutrition Coordinating Center, University of Minnesota, Minneapolis, MN)^14^. The NDSR is based on the USDA data. Thus, the Brazilian foods that were not included in the program database had their nutritional value inserted or adapted from a food of the same value according to national information. The supplement database was analyzed using the Dietary Supplement Assessment Module (DSAM) from NDSR, an NCC enhanced version of the most currently available National Health and Nutrition Examination Survey (NHANES) Dietary Supplement Database 2011-2012. Dietary supplements not found within the current database of the program were added to better reflect the Brazilian marketplace^14^.

### Operational Definitions

Participants were categorized into four groups, according to the use of supplements and the source of the nutrient for usual intake:

1. Non-users: usual nutrient intakes from food by subjects who did not use dietary supplements;
2. Users-foods: usual nutrient intakes from food by consumers of dietary supplements;
3. Users-total: usual total nutrient intakes (from food and dietary supplements) by consumers of dietary supplements;
4. All: non-users and users-total (total sample).

### Statistical Analyses

Usual dietary intake from food and supplement sources was estimated using the Software for Intake Distribution Estimation (PC-SIDE, version 2.0, 2017; Department of Statistics, Iowa State University, Ames, IA, USA). PC-SIDE uses the method proposed by Iowa State University (ISU), which fits a measurement error model to estimate usual dietary intakes, correcting the within-person variance of intake^15^. This method allows initial adjustments for confounding factors, such as day of the week, month, interview mode, or interview sequence, and uses a power transformation to approach the distribution of the observed data closer to normality^15^.

Accordingly, the method used for the supplement analyses was to add the daily nutrient consumption from food and supplements for each day of intake and each individual and then adjust the data using the PC-SIDE program to obtain an estimate of the usual total nutrient intake distribution (add and shrink methodology)^5^^,^^8^^,^^9^.

Analyses of dietary intake from food sources only and the procedures to improve the asymmetric distribution found in nutrients such as folate have been described in detail elsewhere^11^. In brief, to adjust the distribution of this nutrient, the accuracy of the Anderson-Darling statistical test was reduced; and the outliers were replaced by the values corresponding to the mean ± 3 SD (standard deviation)^16^.

The prevalence of inadequate micronutrient intake was estimated according to sex using the Estimated Average Requirement (EAR), as set by the United States Institute of Medicine (IOM), and values above the Tolerable Upper Intake Level (UL) were also considered^17^^–^^20^. For these analyses, we also considered the same value of EAR and UL according to sex for the selected nutrients, except for calcium, which we considered the weighted EAR points to permit analysis of the entire sample^21^.

For the analysis, we selected five nutrients (calcium, folate, and vitamins C, D, and E) with the prevalence of intake inadequacy in the population > 10% above the EAR and because they are usually inadequate in the Brazilian diet^22^^,^^23^. Given that the bioavailability of synthetic folic acid used in supplements and food fortification is higher than that of natural food folate, the expression of folate intakes as μg dietary folate equivalents (DFE) was considered. The fortified flour folate values considered were from the USDA Food Composition Databases. We also compared the usual diet only intake with the total intake (food and supplements) according to age and sex. In addition, a comparison was also made of the total mean usual intake between supplement users and non-users according to BMI and physical activity. Individual sample weight was included to obtain the usual intakes and their errors.

The data did not follow a normal distribution; thus, a Wilcoxon Rank Sum Test was used to compare the two samples. The B-Y method was used to determine the critical value of multiple comparison tests as an alternative to the Bonferroni method, which is more conservative in that case^17^. Thus, the critical value performed in the present study was p ≤ 0.02 considering the 10 multiple comparisons performed. For analysis of usual nutrient intake among BMI categories and between PA condition, we conducted a robust ANOVA (ROBUSTREG in SAS^®^) because our data had important extreme response values. The robust ANOVA verifies interactions and differences among the groups (BMI categories and PA) with the Huber function that reduces weight of extreme values. The level of significance considered was 5%.

Database organization and the initial descriptive analyses such as averages, standard deviation, standard errors, histograms, frequency, normality, and Wilcoxon test were calculated using Statistical Analysis System (SAS) software, version 9.4^24^.

## RESULTS

A total of 35% (n = 179 adults) of the participants reported using some dietary supplement, while 65% (n = 327 adults) reported not using any type of dietary supplement. The majority of supplement users consumed some type of vitamin and mineral supplements (55%), followed by protein (44%), omega-3 (21%), creatine (6%), caffeine (5%), and energy (2%) supplements.

Supplement users included 45% (n = 80) men and 55% (n = 99) women, and the mean age was 43 (standard deviation [SD] = 16.4) years. According to the DRI age classification, the prevalence of supplement users was higher in the older group (35%) with the mean age of 62 (SD = 8.6) years; 87% (n = 156) of the supplement users reported participating in some physical activity. Of the supplement users, 37% (n = 67) were overweight and 10% (n = 18) obese. Over half (50.3%) of the supplement users had higher education level (> 15 years of schooling), and 51% had high socioeconomic level (> US$ 1,878.99 income per month *per person*). On the other hand, among non-users of dietary supplements, a total of 42% (n = 136) were men, and 58% (n = 191) were women, the mean age was 39 (SD = 15) years, with 71% (n = 231) practicing physical activity. Of these, 30% (n = 99) were considered overweight and 17% (n = 55) were obese. A total of 47% of the non-users had higher education level, and 55% had high socioeconomic level (Table 1).

**Table 1:** Characteristics of the study subjects. Brazil, 2016–2017.

		Total		Non-users		Users		p
		n = 506		n = 327		n = 179		
		n	%	n	%	n	%	
Sex								0.49
	Men	216	42.7	136	41.6	80	44.7	
	Women	290	57.3	191	58.4	99	55.3	
Age group								0.03^a^
	20–30y	187	36.9	131	40	56	31.3	
	31–50y	175	34.6	115	35.2	60	33.5	
	≥ 50y	144	28.5	81	24.8	63	35.2	
BMI (kg/m²)								0.06
	< 25	269	53.2	175	52.9	94	52.5	
	25–29.9	166	32.8	99	30.3	67	37.4	
	≥ 30	73	14.4	55	16.8	18	10.1	
Physical activity								< 0.0001^a^
	Yes	387	76.5	231	70.6	156	87.2	
	No	119	23.5	96	29.4	23	12.8	
Socioeconomic class								0.51
	A	111	21.9	67	20.5	44	24.6	
	B	272	53.7	181	55.3	91	50.8	
	C-D-E	123	24.3	79	24.2	44	24.6	

a Significantly different at p ≤ 0.05.

The total mean of usual dietary intake was higher for supplement users than non-users for all five nutrients evaluated (Supplementary material). Considering the corrected p-value for multiple comparisons between the mean dietary intake of users and non-users, only calcium did not reach statistical significance (p = 0.03). Calcium had a mean total intake of 1413 mg (standard error [SE] = 30.6) in users and 1343 mg (SE = 18.2) in non-users; folate had a mean total intake of 388 mcg (SE = 8.5) in users and 355 mcg (SE = 5.0) in non-users (p = 0.0007); vitamin C had a mean total intake of 309 mg (SE = 32.9) in users and 230 mg (SE = 18.4) in non-users (p < 0.0001); vitamin D had a mean total intake of 15.4 mcg (SE = 1.7) in users and 6.6 mcg (SE= 0.3) in non-users (p < 0.0001); and vitamin E had a mean total intake of 14.9 mg (SE = 1.4) in users and 8.9 mg (SE = 0.3) in non-users (p < 0.0001).

**Figure 1:**
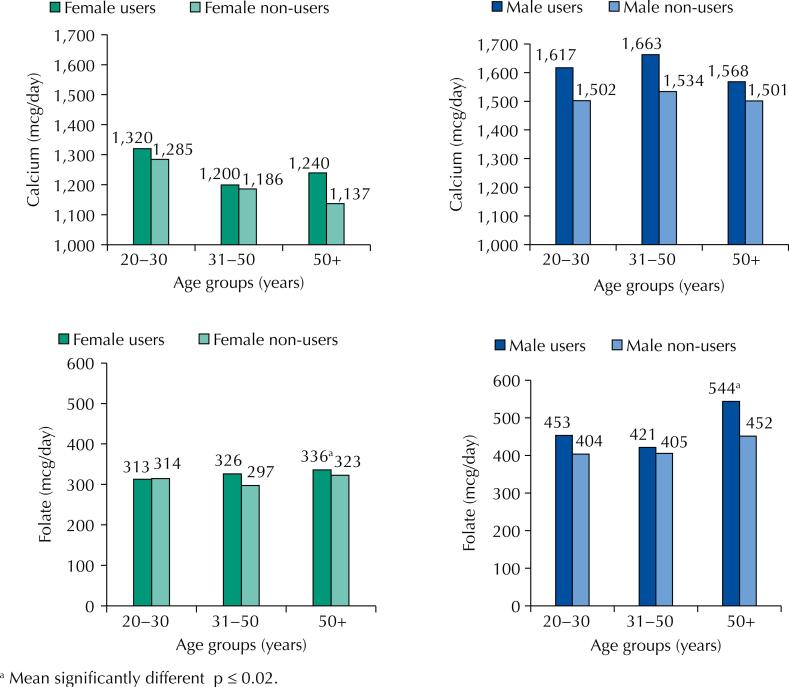
Usual intake of calcium and folate by users and non-users of dietary supplements according to age and sex. Brazil, 2016–2017.

**Figure 2:**
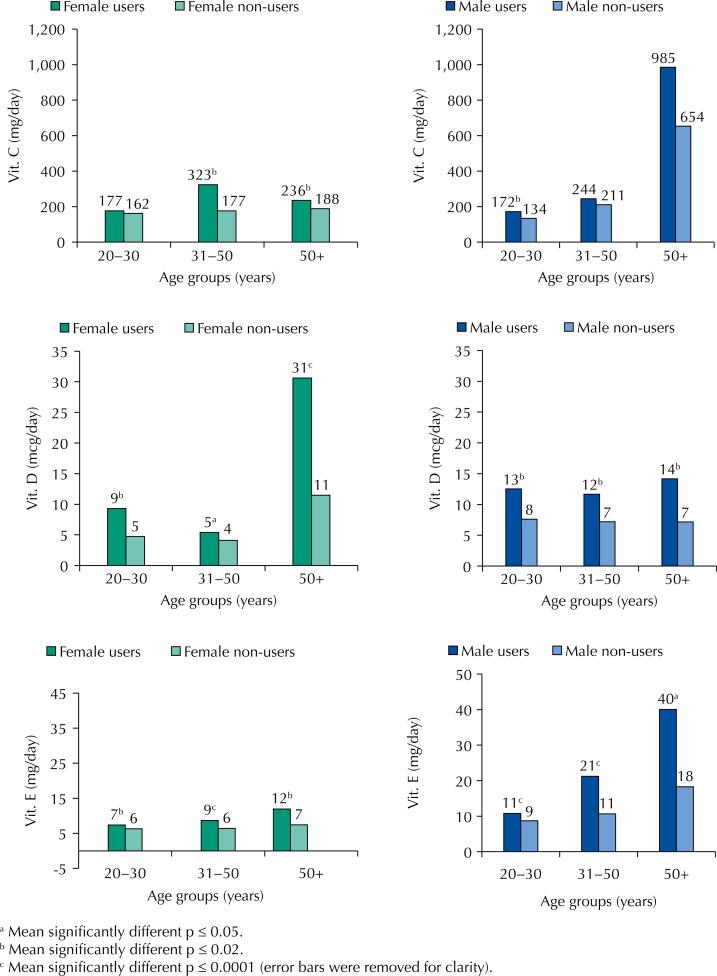
Usual intake of vitamins by users and non-users of dietary supplements according to age and sex. Brazil, 2016–2017.

Figures 1 and 2 show that men had the highest intake in almost all age groups for the five nutrients analyzed, except for vitamin D in women in the 50+ age group. We found differences in age groups with the highest intake of nutrients among users of supplements for men and women.

Dietary supplement use consistently contributed to increasing the intake of nutrients and decreasing population prevalence of inadequacy according to sex (Table 2). The prevalence of inadequacy of non-users is higher than users-total and, among users-foods, this prevalence was higher only for calcium. Only small (typically < 13%) increases in the prevalence of the group exceeding the UL. According to all groups, women had the highest prevalence of inadequacy for calcium, folate, and vitamin D (Table 2). Thus, the prevalence of inadequacy for folate was high in all groups, especially in female non-users, with 63.3% inadequacy. The use of supplements contributed to decreasing the prevalence of inadequacy for folate, but it was still prominent (52% for women). The prevalence of folate inadequacy has internal validity due to differences in food fortification between Brazil and the USA.

**Table 2:** Selected results from the distribution of mean of usual intake and prevalence of inadequate and toxicity of nutrients intake according to sex. Brazil, 2016–2017.

		Non-users				Users-foods				Users-total				All			
		PI		Above ul		PI		Above ul		PI		Above ul		PI		Above ul	
		%	95%CI	%	95%Cl	%	95%Cl	%	95%Cl	%	95%Cl	%	95%Cl	%	95%Cl	%	95%Cl
n		327				179				179				506			
Calcium (mg)																	
	Men	2.3	2.2–2.4	2.2	2.1–2.3	2.9	2.8–3.0	6.1	6.0–6.2	1.4	1.4–1.4	12.6	12.5–12.7	2.5	2.5–2.5	7.45	7.4–7.5
	Women	13.2	13.1–13.3	0.2	0.2–0.2	18.8	18.7–18.9	0.2	0.2–0.2	5.8	5.6–6.0	0.1	0.1–0.1	12.3	12.2–12.4	2.2	2.2–2.2
Folate (mcg)																	
	Men	26.6	26.4–26.8	0	0	19.5	19.3–19.7	0.4	0.4–0.4	7.4	7.2–7.6	10.6	10.4–10.8	19.4	19.3–19.5	0.8	0.8–0.8
	Women	63.3	63.3–63.4	0	0	56.2	56.0–56.4	0	0	25.8	25.5–26.1	0	0	52.1	52–52.2	0	0
Vitamin C (mg)																	
	Men	27.5	27.4–27.6	1.5	1.5–1.5	13.1	12.8–13-4	0	0	3.5	3.3–3.7	0	0	18.6	18.5–18.7	1.9	1.9–1.9
	Women	22.2	22.1–22.3	1.8	1.8–1.8	11.6	11.4–11.8	0.5	0.5–0.5	3.9	3.8–4.0	0	0	15.1	15.0–15.2	0.6	0.6–0.6
Vitamin D (mcg)																	
	Men	96.9	96.8–97	0	0	84.7	84.5–84.9	0	0	32.2	32–32.4	2.4	2.3–2.5	64.1	64.0–64.2	0.4	0.4–0.4
	Women	96.6	96.5–96.7	0	0	98.9	98.9–98.9	0	0	34.8	34.6–35.0	4.7	4.6–4.8	68.3	68.2–68.4	1.9	1.9–1.9
Vitamin E (mg)																	
	Men^a^	93.9	93.8–94	0	0	80.3	80.1–80.5	0	0					58.9	58.8–59.0	0	0
	Women	99.5	99.5–99.5	0	0	98.2	98.1–98.3	0	0	54.0	53.9-54.1	0	0	81.7	81.6–81.8	0	0

PI: prevalence of inadequacy; uI: tolerable upper intake levels.

a Asymmetrical distribution.

For vitamin C, there was a 10% and 8% difference between the prevalence of inadequacy for users-foods and users-total for men and women, respectively (Table 2). Only the top 5% (P95) and 10% (P90) of men met the EAR for vitamin D and E through the diet, respectively. When dietary supplement use was included, the prevalence of inadequacy was dramatically lower with > 40% of the difference between users-foods and users-total (Table 2).

Regarding BMI categories and use of supplements, we observed significant interactions for these conditions for almost all nutrients, except for vitamin D. Usual intake of vitamin D was significantly higher for supplement users when compared to non-users (p < 0.0001) (Table 3). Concerning physical activity, we found a significant interaction between physical activity and the use of supplements for vitamin D and calcium. In contrast, vitamin C presented a non-significant interaction but a significant difference for non-users compared with users of supplements (p < 0.00001) (Table 3).

**Table 3:** Distribution of means of usual micronutrients intake according to body mass index (BMI) and physical activity. Brazil, 2016–2017.

Nutrients	BMI < 25kg/m²		BMI 25–29.9kg/m²		BMI > 30kg/m²		p	Physical activity		Physical inactivity		p^b^
	94	173	67	99	18	55		156	231	23	96	
	Users	Non-users	Users	Non-users	Users	Non-users		Users	Non-users	Users	Non-users	
Calcium (mg)^a^	1,469.4±393.8	1,263.2±357.2	1,408.5±451.3	1,380±297.7	1,159.9±345.3	1,289.7±385.7	0.0029	1,605.1±870.2	1,340.8±387.7	1,185.4±288.1	1,218.3±315	0.0212
Folate (mcg)^a^	521.5±692.2	334.6±108.3	711.5±545.9	356.9±73.4	347.1±127.7	318.9±69.1	0.0034	602.7±1,155.3	346.8±102.3		319.2±74.1	
Vitamin C (mg)^a^	294.7±152.3	453.2±1,898.1	279.7±274.7	216.4±382.4	377.4±566.9	291.4±529.1	0.0001	278.1±192.4	222.6±387.4	334.3±323.3	968±7,003.0	0.3687^c^
Vitamin D (mcg)^a^	34.9±95.2	4.6±3.4	28.9±59.8	4.5±1.8		4.2±1.9	0.1454^c^	22.6±46.4	4.7±2.7	27.9±19.8	3.8±1.4	< 0.0001
Vitamin E (mg)^a^	21.8±24.5	6.6±2.2	15.7±9.8	6.9±1.4	144.3±171.1	10.9±3.3	0.0001	21.2±18.6	7.0±2.4		5.9±1.7	

a Mean ± standard error.

b Significantly different at p ≤ 0.05 using robust ANOVA for the interactions of BMI categories x supplements and physical activity x supplement.

c Significant different for supplement p < 0.00001.

## DISCUSSION

The assessment of dietary intakes from supplement sources must be included to characterize the total exposure to nutrients and estimate the total usual nutrient intake distribution. The inclusion of dietary supplements prevents the mean nutrient intake and the prevalence of excessive intake from being underestimated and the prevalence of inadequate intake from being overestimated^25^.

Dietary supplement users are more likely than non-users to adopt positive health-related habits^2^^,^^3^^,^^26^. The differences in the levels of the nutrients between supplement users and non-users are not very high but consistent, indicating that supplement use is part of an overall approach to healthy living^3^^,^^26^. However, dietary supplement use can also increase the risk of intakes above the tolerable upper intake level (UL), which can cause undesirable side effects^4^^,^^25^.

We found that dietary supplement users were more prevalent among women, in the older age group, physically active, with a BMI < 25kg/m² and tended to have higher usual mean intakes of nutrients from their diets than non-users, especially in men. Similar results were also found in European countries^3^^,^^27^^,^^28^, in the USA^2^^,^^6^ and Canada^29^, and a sample of individuals in the city of São Paulo in Brazil^7^.

Our analysis shows that dietary supplements significantly increased nutrient intake and decreased the prevalence of inadequacy for most nutrients. These data also show that nutrient intake from fortification and dietary supplements do not present a meaningful risk for overconsumption as UL was very low. Our findings of lower prevalence of inadequacy with supplement use are similar to those reported from the National Health and Nutrition Examination Surveys (NHANES)^30^, Continuing Survey of Food Intakes by Individuals (CSFII)^15^, and the Hawaii-Los Angeles Multiethnic Cohort (MEC)^25^. Thus, it seems that supplements had a positive influence on nutrient adequacy.

In our results, female users had a difference of 13% lower prevalence of calcium inadequacy from the total intake (diet + supplements) than with calcium intake from diet only, and had higher vitamin D intake than men among users and non-users. The increased rates of supplement usage among women could also be partially attributed to the increased use of supplemental calcium and vitamin D among women to maintain bone health throughout the lifespan and prevent the onset of osteoporosis during aging^31^.

The fortification of food supply with folic acid contributes to enhance the diet of reproductive-age women and prevents neural tube effects (NTD). In this study, among supplement users, 44% of women meet the EAR through the diet alone and considering dietary supplements and foods, 74% meet the EAR. Data from the Brazilian National Food Survey (INA) indicate that it is almost impossible for women of childbearing age to achieve the recommended additional folate intake only from food sources of natural folate, as these women would need to drastically increase their intake of fruit, vegetables, legumes, and pulses in their diet. Even considering the intake of fortified food products, the recommended level of folate is still not reached in Brazil^32^. Also, our study describes the intake among users and non-users of dietary supplements.

Interestingly, we found a slightly higher folate intake for non-users of supplements in women of childbearing age. These results suggest that instituted national mandatory fortification programs with folic acid to enhance the diets of reproductive-age women may be partially increasing the dietary folate intake^33^. Therefore, careful monitoring of total folate intake from food and dietary supplements is recommended^23^^,^^34^^,^^35^.

We also observed that older adults had better vitamin C and E status partially due to supplements. Vitamin C and E are antioxidants that presumably have a protective effect by either reducing or preventing oxidative damage associated with aging^36^. Their antioxidant properties may also explain the high intake of vitamin C and E in physically active individuals. Some studies indicate that users of dietary supplements are more likely to engage in sports activity than non-users^2^^,^^3^.

In public health nutrition research, the distribution of usual total intake is a necessary cornerstone. Furthermore, we must address the complex relationships between nutrient intake from food and from dietary supplements. Thus, we believe this is the first Brazilian study to describe the intake among users and non-users of dietary supplements. It is also important to note that several other studies used frequency of intake to collect the supplement intake rather than the daily intake^8^^,^^28^^,^^30^^,^^31^, which does not estimate supplement day-to-day variability^9^. Therefore, the strength of this paper is the estimate and the distribution adjusted to reflect total usual nutrient intake using the ISU method, because we collected intake of dietary supplements as part of the 24-HR.

Methodological limitations demand caution in interpreting the results from this research. The study was a cross-sectional survey, which prevents directional conclusions or causality; supplement use is subject to misreporting; few skewed distributions with a small number of subjects did not allow adjustment of the analysis to converge. Also, the nutrient content of dietary supplements is based on label values that could exceed actual amounts. Despite the corrections made in the database, the values obtained for all minerals may contain inaccuracies, especially given the differences between food fortification in the USDA table and the Brazilian food tables. Nonetheless, the usual total intake distributions are necessary to accurately monitor the population's nutritional status and compliance with reference intake values.

In conclusion, we found that dietary supplements contributed to increasing the prevalence of nutrient adequacy in the diet, with a low percentage of the population exceeding the tolerable intake limits. The study also allows relating the intake of food/ supplements to the practice of physical activity that may be discretionary for the guidance of physically active individuals who are known to be more prone to dietary supplements. Moreover, the data presented in our study shows that through appropriate choices of methods to evaluate the distribution of nutrients intake, it is possible to face the difficulties involved by the high degree of complexity in the measurement and analysis of dietary intake.
